# Motor Mechanism for Protein Threading through Hsp104

**DOI:** 10.1016/j.molcel.2009.02.026

**Published:** 2009-04-10

**Authors:** Petra Wendler, James Shorter, David Snead, Celia Plisson, Daniel K. Clare, Susan Lindquist, Helen R. Saibil

**Affiliations:** 1 Department of Crystallography, Birkbeck College, Malet Street, London WC1E 7HX, UK; 2 Department of Biochemistry and Biophysics, University of Pennsylvania School of Medicine, 422 Curie Boulevard, Philadelphia, PA 19104-6059, USA; 3 Howard Hughes Medical Institute, Department of Biology, Massachusetts Institute of Technology and Whitehead Institute for Biomedical Research, Nine Cambridge Center, Cambridge, MA 02142, USA

**Keywords:** PROTEINS

## Abstract

The protein-remodeling machine Hsp104 dissolves amorphous aggregates as well as ordered amyloid assemblies such as yeast prions. Force generation originates from a tandem AAA+ (ATPases associated with various cellular activities) cassette, but the mechanism and allostery of this action remain to be established. Our cryoelectron microscopy maps of Hsp104 hexamers reveal substantial domain movements upon ATP binding and hydrolysis in the first nucleotide-binding domain (NBD1). Fitting atomic models of Hsp104 domains to the EM density maps plus supporting biochemical measurements show how the domain movements displace sites bearing the substrate-binding tyrosine loops. This provides the structural basis for N- to C-terminal substrate threading through the central cavity, enabling a clockwise handover of substrate in the NBD1 ring and coordinated substrate binding between NBD1 and NBD2. Asymmetric reconstructions of Hsp104 in the presence of ATPγS or ATP support sequential rather than concerted ATP hydrolysis in the NBD1 ring.

## Introduction

Members of the AAA+ (ATPases associated with various cellular activities) super family of protein-remodeling factors employ the energy of ATP binding and hydrolysis to dissolve amorphous or amyloid aggregates. In contrast to structurally well characterized, ATP-fueled machines such as GroEL, kinesin, and myosin, the mechanisms by which the wide variety of AAA+ enzymes convert chemical energy into mechanical movement are poorly understood (reviewed in Vale, 2000). The common feature of the AAA+ super family is a conserved nucleotide-binding module that can be equipped with a multitude of accessory motifs and domains conferring different substrate specificities and functions (reviewed in Hanson and Whiteheart, 2005; Erzberger and Berger, 2006). Nucleotide-dependent conformational changes have been observed for several AAA+ proteins (Bochtler et al., 2000; Wang et al., 2001; DeLaBarre and Brunger, 2005; reviewed in Pye et al., 2006; Gai et al., 2004). AAA+ proteins only act efficiently in oligomeric assemblies. It is thought that ATP binding and hydrolysis evoke small movements between the AAA+ subdomains that allow the active hexamer to perform processive mechanical work on the substrate (Gai et al., 2004). In the case of adaptor-bound p97 (Beuron et al., 2006) and SV40 large tumor antigen helicase (Gai et al., 2004), a concerted activity of all subunits has been suggested, but some crystal structures of AAA+ proteins and strong evidence from biochemical experiments on ClpX, PAN, and MCM helicase complexes indicate sequential or probabilistic processivity in the superfamily (DeLaBarre and Brunger, 2005; Singleton et al., 2000; Martin et al., 2005; Hersch et al., 2005; Horwitz et al., 2007; Moreau et al., 2007).

The protein-remodeling factor Hsp104 (heat shock protein 104) contains two nucleotide-binding domains (NBDs) per subunit. Kinetic studies to unravel the contribution of each ATPase domain to Hsp104's catalytic activity revealed cooperativity between AAA+ domains within rings and subunits (Schirmer et al., 2001; Hattendorf and Lindquist, 2002; Schaupp et al., 2007). Owing to the complexity of the two-tiered hexamer, a structural description of the allosteric interactions is still lacking. It is known that NBD1 provides the main hydrolytic activity of Hsp104, although it shows lower affinity for ATP than does the C-terminal NBD, which promotes nucleotide-dependent hexamerization (Hattendorf and Lindquist, 2002; Schirmer et al., 1998). ATP binding to NBD2 increases the activity of NBD1 (Doyle et al., 2007), and keeping NBD1 bound to ATP greatly stimulates ATP hydrolysis in the otherwise barely active NBD2 domain (Schaupp et al., 2007). As in many AAA+ proteins, substrate interaction occurs in the ATP-bound state, and ATP occupancy in NBD1 has recently been shown to be essential for high-affinity binding of substrate to Hsp104 (Bosl et al., 2005; Schaupp et al., 2007). So far, the only two substrate recognition sites of Hsp104 are ascribed to an α-helical insertion in NBD1 and a β-hairpin in NBD2, located before helix α2 in the αβ subdomain in both NBDs (Lum et al., 2004; Lum et al., 2008). Mutations of conserved tyrosine residues within these motifs strongly impair refolding activity in vitro (Lum et al., 2008), and especially mutations in NBD2 result in severe loss of thermotolerance in vivo (Lum et al., 2004).

We have shown that Hsp104 hexamers bound to ATPγS adopt an AAA+ domain arrangement that is incompatible with the classical hexameric packing (Wendler et al., 2007). The ClpB/Hsp104 subgroup has a characteristic coiled-coil insertion that emerges from the α-helical subdomain of NBD1. In our structures, this coiled coil is suggested to intervene in subunit packing by covering the cavity-facing side of NBD1 and interacting with NBD2. Comparison of N-terminally truncated Hsp104 (ΔN Hsp104) and full-length Hsp104 N728A (sensor1) in the presence of ATPγS (Wendler et al., 2007) does not show any structural differences attributable to the sensor 1 mutation in NBD2, qualifying this mutant as a good candidate for structural studies. The Hsp104^N728A^ mutation allows binding, but not hydrolysis, of ATP in NBD2 and enables us to control the hydrolysis state in one domain, stalling at least one AAA+ ring in a defined nucleotide state.

## Results

In order to understand the conformational changes upon ATP binding and hydrolysis within Hsp104 hexamers, we pursued two cryoelectron microscopy (cryo-EM) strategies. First, we determined the 3D structures of Hsp104^N728A^ hexamers in the presence of ADP and ATP and compared them to the structure in the presence of ATPγS. Second, we generated asymmetric reconstructions of the ATPγS and ATP Hsp104 hexamers and analyzed the domain orientations of individual subunits.

### Comparison of Pore Sizes in Hsp104^N728A^ ATP, ADP, and ATPγS Hexamers

**Figure 1:**
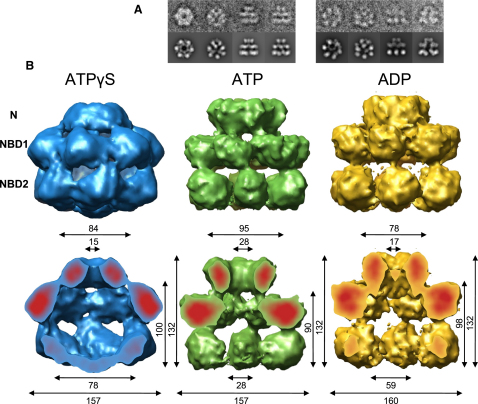
Cryo-EM Reconstructions of Hsp104^N728A^ in the Presence of ATPγS, ATP, and ADP (A) (Upper row) Class averages generated from the final data set obtained by multivariate statistical analysis in IMAGIC, containing an average of 15 images per class. (Lower row) Corresponding reprojections of the 3D structures. (B) Three-dimensional reconstructions of Hsp104^N728A^ in the presence of ATPγS (blue), ATP (green), and ADP (yellow) as side view (upper row) and cut-open side view (lower row). Surface views show the density rendered at a threshold accounting for a molecular mass of 612 kDa. Red areas of the cut surfaces indicate high-density core regions. Measurements are given in Å.

We collected data sets of 4046 and 2379 particles for Hsp104^N728A^ in the presence of ATP and ADP and generated independent 3D reconstructions by angular reconstitution with 6-fold symmetry. Refinement yielded maps with good agreement between input class averages and corresponding reprojections (Figure 1A) and ∼12 Å resolution (Figure S1 available online).

Both maps have overall dimensions of ∼160 Å × 130 Å (Figure 1B), very similar to the previously obtained Hsp104 ^N728A^ ATPγS map (Wendler et al., 2007). In all nucleotide states, the hexamers assemble into N-terminal, NBD1, and NBD2 rings, as determined by comparison with Hsp104 ΔN ATPγS. The handedness of Hsp104-ATP and Hsp104-ADP was chosen so that the core densities of the domains match when the maps are overlaid with the Hsp104 ^N728A^ ATPγS map (handedness determined in Wendler et al., 2007).

The N-terminal rings of all three reconstructions account for six Hsp104 N-terminal domains (108–115 kDa) at surface thresholds enclosing a molecular mass of 612 kDa for each hexamer, but they differ considerably in outer diameter (78–95 Å) and width (15–28 Å) of the central pore. For the NBD1 ring, the outer diameter is identical in all three reconstructions, but the enclosed central cavity ranges from 28 Å in the ATP hexamer to 78 Å in the ATPγS complex. The hexamer with the largest pore in the N-terminal ring shows the smallest pore in the NBD1 ring (ATP), and, conversely, the hexamer with the smallest pore in the N-terminal ring shows the largest pore in the NBD1 ring (ATPγS). The NBD2 densities of the ATP and ADP hexamers are less well defined, hardly connected, and form an open ring structure in contrast to the ones in the ATPγS hexamer. They also lack the central density at the base of the cavity, which has been attributed to the C-terminal region of Hsp104 in the ATPγS state. Despite a similar overall height of the reconstructions, the double tier of the hexamer in the presence of ATP is about 10 Å shorter than the others.

### Hsp104 Shows Large, Nucleotide-Dependent Domain Movements

Due to the Hsp104^N728A^ mutation, NBD2 should be either ATP bound or empty in the presence of ATP or ATPγS. Because ATP binding to NBD2 is necessary for oligomerization of Hsp104, we assume that most NBD2 domains are nucleotide bound. NBD1, on the other hand, is able to hydrolyze ATP and can, therefore, also be captured in the ADP-bound state when ATP is present. Considering that the N728A mutant maintains 25% of the wild-type ATP turnover rate at NBD1 (Hattendorf and Lindquist, 2002), it is likely that we are observing the posthydrolysis state (ADP or ADP.Pi) in the active, NBD1 ring, while NBD2 remains ATP bound in the presence of ATP. To indicate the nucleotide status of the domains in the different maps, we refer to the states as T/T (in the presence of ATPγS), D/T (in the presence of ATP), and D/D (in the presence of ADP).

**Figure 2:**
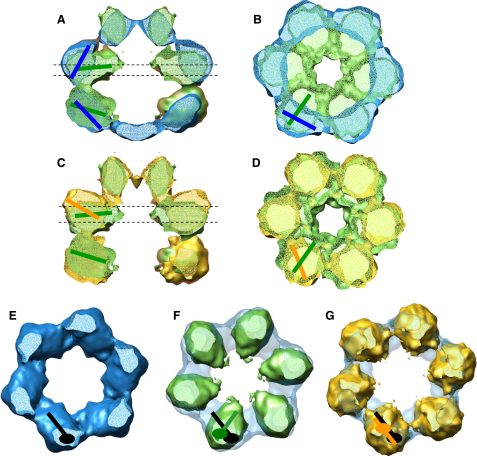
Analysis of Nucleotide-Dependent Domain Movements (A) A 15 Å section through the overlaid side view sections of Hsp104^N728A^ ATPγS and ATP. Colored lines indicate the long axes of the domains. (B) Overlaid NBD1 cross-sections of Hsp104^N728A^ ATPγS and ATP. Section position is indicated by dashed lines in (A). Colored lines indicate the long axes of the domains. (C) A 15 Å section through the overlaid side views of Hsp104^N728A^ ATP and ADP. Colored lines indicate the long axes of the domains. (D) Overlaid NBD1 cross-sections of Hsp104^N728A^ ATP and ADP. Section position is indicated by dashed lines in (C). Colored lines indicate the long axes of the domains. (E) Cross-section of Hsp104^N728A^ ATPγS NBD2. The position of the connection to NBD1 is indicated by a black ellipse. The long axis of the domain is represented by a black line. (F) Overlaid NBD2 cross-sections of Hsp104^N728A^ ATPγS and ATP. The connection to NBD1 and the long axis of the domain are indicated as a green ellipse and line. Black line and ellipse are as in (E). (G) Overlaid NBD2 cross-sections of Hsp104^N728A^ ATPγS and ADP. The connection to NBD1 and the long axis of the domain are indicated as an orange ellipse and line. Black line and ellipse are as in (E).

Even though the outer dimensions hardly vary between the three reconstructions, the domain arrangement within the layers is strikingly different. Between the T/T and D/T states, the N-terminal end of NBD1 rotates into the cavity, narrowing the central pore in this ring to 28 Å in D/T (Figures 2A, 2B, and 1B). Consequently, the height of the double layer and the distance between the core densities of the two NBDs is reduced by ∼10 Å. The bottom of the N-terminal domain is pulled inward by the movement of NBD1 so that the cone-shaped N-terminal ring observed in T/T becomes funnel shaped in the presence of ATP (D/T). The movement of NBD1 also causes an in-plane rotation of the inactive NBD2 around its connection to NBD1, thereby disrupting the interdomain contacts in this ring (Figures 2E and 2F).

In the presence of ADP (D/D), the contacts between adjacent AAA+ domains are weak in both layers, whereas the N-terminal domains remain closely joined. The N-terminal domains, which are pulled slightly into the cavity, have a parallel alignment with an ∼17 Å central channel opening (Figure 1B). Judged by the connection to the N termini, the width of the central cavity in NBD1, and the orientation of the core densities, the N-terminal end of NBD1 seems to move downward and outward in the presence of ADP (Figures 2C and 2D, orange lines). NBD2 appears rotated by almost 180° about the connection to NBD1 between the D/D and T/T states, judged by the position of the long axis of the domain and the connection to NBD1 (Figure 2G). Thus, during the transition from T/T to D/T to D/D, ATP hydrolysis in each domain appears to trigger a 90° rotation in NBD2.

### Domain Fitting Indicates the Position of Substrate-Binding Loops during ATPase Cycle

**Figure 3:**
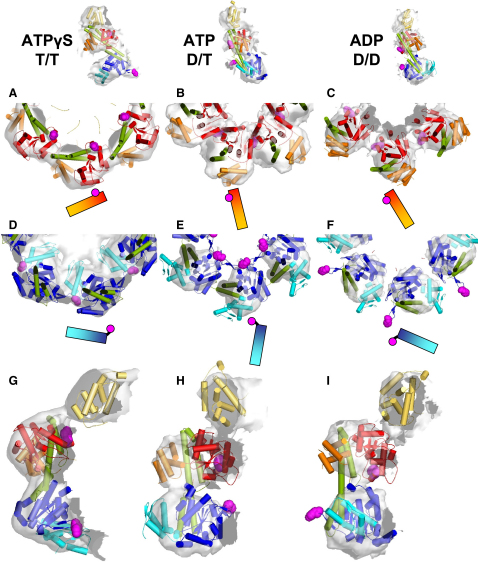
Domain Docking of the Hsp104 Homology Model into EM Densities Rigid body domain fitting into the Hsp104^N728A^ ATPγS (A, D, and G), ATP (B, E, and H), and ADP (C, F, and I) maps. Domains and subdomains are color-coded as follows: N, yellow-orange; NBD1, red/orange; NBD2, blue/cyan; coiled coil, green. The ATP-binding pocket is located at the interface between the NBD subdomains (red/orange, blue/cyan). The ClpB X-ray structure (PDB code:1QVR) was used for docking into the N-terminal density. Tyrosines 257 and 662 are depicted as magenta spheres. Cross-sections through NBD1 of Hsp104^N728A^ ATPγS (A), ATP (B), and ADP (C), showing half of a ring. Equivalent views of NBD2 of Hsp104^N728A^ ATPγS (D), ATP (E), and ADP (F). The long axis of the domain and positioning of Tyr 257 and Tyr 662 are indicated in cartoons. Side view sections of an Hsp104^N728A^ ATPγS subunit (G), ATP (H), and ADP (I) subunit.

The nature of the domain movements in Hsp104 is best seen in movies that show the maps obtained for the different nucleotide-bound states in alternation (Supplemental Data). In order to provide a better description of the nucleotide-dependent changes, we interpreted the domain movements by fitting the N, NBD1, coiled-coil, and NBD2 domains independently as rigid bodies into the density maps (Figure 3). We used a fit for the Hsp104 ATPγS map as a starting point and considered the electron microscopy (EM) densities and the steric feasibility of the domain movement to guide the docking. We also restricted the rotational freedom between NBD1 and NBD2 to ∼30° because several findings indicate that allosteric communication is accomplished via the short linker (10 amino acids) between the AAA+ domains. For instance, mutation of the conserved proline residue in this region leads to loss of disaggregation activity toward certain substrates (Kurahashi and Nakamura, 2007), and complex formation of noncovalently linked NBD1 and NBD2 can be obtained when NBD2 is extended N terminally by 15 amino acids to include the last α helix of NBD1, suggesting a coupling rigid enough to transmit allosteric signals (Beinker et al., 2005). With regard to the connecting density and the distance between NBD1 and NBD2, the subunit placement fits the densities of all three maps best when the AAA+ domains are connected as shown in Figure 3. The ATPγS fit for the N-terminal domain and NBD1 (Wendler et al., 2007) has been slightly adjusted, and the domain connectivity with NBD2 has been switched to the adjacent subunit in light of the results presented here (Figure S2).

When we interpret the domain rotations on the basis of the fits, the most obvious movements occur upon ATP hydrolysis in NBD1 (comparison between T/T and D/T states). Though both NBDs experience a 90° rotation in the plane of the ring (NBD1: Figures 3A and 3B; NBD2: Figures 3D and 3E), they also show an out-of-plane rotation downward (NBD1) and upward (NBD2) into the cavity (Figures 3G and 3H). Consequently, the N-terminal α/β subdomains of both NBDs point into the cavity in the presence of ATP (D/T) (Figures 3B and 3E). The solid connection between the two AAA+ rings and unfilled density in both domains after docking of the NBDs leads us to suggest that the coiled-coil domain stays intercalated between the NBDs. Such location of the coiled coil (∼94 Å length) is compatible with the 90–100 Å height of the Hsp104 double tier compared to 80–90 Å in EM reconstructions of ClpA and p97 (Ishikawa et al., 2004; Rouiller et al., 2002) and 70 Å for the AAA+ double layer in the p97 X-ray structure (DeLaBarre and Brunger, 2003). The densities indicate that the coiled-coil domain does not rigidly follow the movement of NBD1 (Figures 3A, 3B, 3G, and 3H), arguing against our earlier proposal that the conserved tyrosine 507 in the mutational hotspot on the coiled coil might participate directly in substrate translocation (Wendler et al., 2007). In the ADP complex (D/D), subunit contacts are weak, allowing greater mobility of the domains and resulting in less featured density than in the other maps so that fitting is less reliable. The AAA+ domains were fit to match the long axes of the densities, with the coiled coil intercalated between the AAA+ domains as for the D/T state. The lack of defined density could indicate that the coiled coil disconnects from the AAA+ domains and that the subunit adopts a conformation similar to the one seen in the ClpB X-ray structures (Lee et al., 2003). Relative to the D/T state, both domains experience a further in-plane rotation (NBD1: Figures 3B and 3C; NBD2: Figures 3E and 3F), causing the interdomain connections within the rings to break up and placing the coiled coil on the outside of the complex (Figures 3C and 3F).

From related AAA+ hexamers ClpA and ClpX, it is known that several substrate-binding loops are required for efficient substrate remodeling (Hinnerwisch et al., 2005; Martin et al., 2008). However, in Hsp104, tyrosine motifs in each AAA+ domain (NBD1-Tyr: K^256^YKG^259^; NBD2-Tyr: G^661^YVG^664^) are the only loops so far known to be involved in substrate binding and translocation (Lum et al., 2004, 2008). Thus, it is particularly interesting to track their positions through the different nucleotide states as a marker for substrate binding. Although the secondary structures of these motifs are undefined due to disorder in the X-ray structures, we can assign their approximate locations on the NBDs in the Hsp104 homology model (Figure 3). NBD1-Tyr is positioned on the N-terminal rim of the cavity-facing side of NBD1 in the T/T state (Figure 3G) and is shifted ∼25 Å inward and ∼15 Å down toward the center in the D/T structure (Figure 3H), where it merges into the interface with the neighboring subunit close to the 28 Å pore formed by the NBD1 ring (Figure 3B). ATP hydrolysis in NBD1 triggers a rotation of the inactive NBD2, which moves NBD2-Tyr from the interface with the right-hand neighbor by ∼20 Å toward the N-terminal end of the central pore in the second AAA+ ring (Figures 3D and 3E). In the presence of only ADP, the fit suggests that both Tyr motifs are rotated away from the cavity (Figures 3C and 3F).

### Hsp104 NBD2 Tyr Motif and Coiled Coil Are Shielded in the Presence of ATPγS

**Figure 4:**
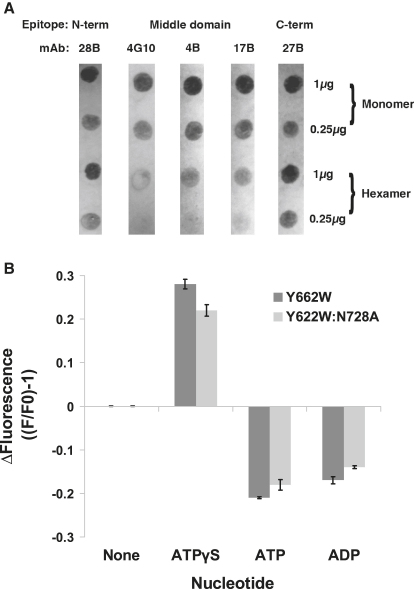
Surface Exposure of Hsp104 Coiled-Coil Domain and NBD2 Tyr Loops (A) Monomeric or hexameric Hsp104 (1 μg or 0.25 μg) was applied to nitrocellulose and probed with monoclonal antibodies that recognize either the N-terminal domain (28B), middle domain (4G10, 4B, and 17B), or C-terminal domain (27B) of Hsp104. (B) Hsp104^Y662W^ or Hsp104^Y662W:N728A^ (2 μM) was incubated in the absence or presence of ATPγS, ATP, or ADP (1 mM) for 10 min at 25°C. The change in tryptophan fluorescence (ΔF) was determined by comparing the fluorescence after 10 min (F) to the starting fluorescence (F_0_), such that ΔF = (F/F_0_) − 1. Values represent means ± SD (n = 4).

The fits deduced from the EM densities were probed by biochemical analyses examining surface accessibility by antibodies and conformational changes in NBD2 by tryptophan fluorescence measurements. To assess epitope accessibility in monomeric and hexameric Hsp104 (ATPγS), we prepared dot blots with monoclonal antibodies (mAbs), which recognize the N-terminal domain, the coiled coil, and the C-terminal domain of Hsp104 (Cashikar et al., 2002). Strikingly, all mABs that recognize the coiled-coil domain bind monomeric Hsp104 better than hexameric T/T Hsp104, whereas mAbs that recognize the N- or C-terminal domain bind monomers and hexamers equally well (Figure 4A). The previously observed stimulation of Hsp104 ATPase activity by coiled-coil mAbs (Cashikar et al., 2002) might thus stimulate hydrolysis in monomers rather than hexamers, possibly by retaining them in a favorable conformation or by transient or substoichiometric binding to hexamers.

Conformational changes in NBD2 were measured by nucleotide-dependent changes in fluorescence with the single tryptophan mutants Hsp104 Y662W and Hsp104 Y662W:N728A (Figure 4B). In comparison to the intrinsic fluorescence of Hsp104 hexamers in the absence of nucleotide, fluorescence emission of hexamers increases significantly in the presence of ATPγS and is quenched in the presence of ATP and ADP. The results are similar for wild-type and Hsp104^N728A^. They resemble the data obtained by Lum et al. (2004) under slightly different conditions and extend their study by analyzing tryptophan fluorescence in the presence of ATPγS. Consistent with our fits deduced from EM densities, these data indicate that W662 moves into a more hydrophobic environment in the presence of ATPγS and a more hydrophilic environment in the presence of ATP or ADP.

### Hsp104-ATPγS Is Strongly Asymmetric

**Figure 5:**
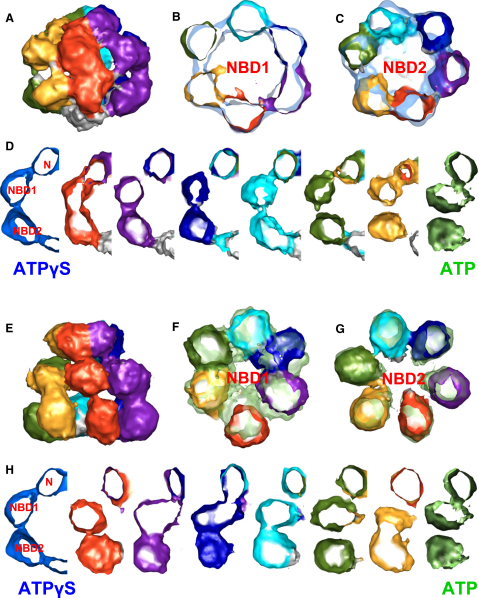
Asymmetric Cryo-EM Reconstructions of Hsp104 ATPγS and ATP (A) Side view of the asymmetric 3D reconstruction of Hsp104^N728A^ ATPγS. Each subunit is colored differently. (B) Overlaid cross-sections through NBD1 of 6-fold (transparent blue) and asymmetric maps of Hsp104^N728A^ ATPγS. Color code of the asymmetric reconstruction is as in (A). (C) Overlaid cross-sections through NBD2 of 6-fold and asymmetric maps of Hsp104^N728A^ ATPγS. Color code of the asymmetric reconstruction is as in (A). (D) Side view section through each subunit of the asymmetric reconstruction compared to sections of 6-fold symmetric Hsp104^N728A^ ATPγS (left) and Hsp104^N728A^ ATP (right). N, NBD1, and NBD2 indicate domain assignments. (E) Side view of the asymmetric 3D reconstruction of Hsp104^N728A^ ATP. Each subunit is colored differently. (F) Overlaid cross-sections through NBD1 of 6-fold (transparent green) and asymmetric maps of Hsp104^N728A^ ATP. Color code of the asymmetric reconstruction is as in (A). (G) Overlaid cross-sections through NBD2 of 6-fold and asymmetric maps of Hsp104^N728A^ ATP. Color code of the asymmetric reconstruction is as in (A). (H) Side view section through each subunit of the asymmetric reconstruction compared to sections of 6-fold symmetric Hsp104^N728A^ ATPγS (left) and Hsp104^N728A^ ATP (right). N, NBD1, and NBD2 indicate domain assignments.

The processivity of the disaggregation reaction of Hsp104 is unknown, but studies on single AAA+ hexamers such as ClpX and MCM helicase suggest a random rather than concerted or strictly sequential ATP hydrolysis model (Martin et al., 2005; Moreau et al., 2007). To investigate the possibility of variable conformations in individual Hsp104 subunits, we increased the ATP and ATPγS data sets to 11,128 and 23,632 particles, respectively, and refined the maps without symmetry (Figure 5). The resulting reconstructions resembled the symmetrized maps in overall dimensions and had 14.1 Å and 11.5 Å resolutions at 0.5 Fourier shell correlation (FSC) (Figure S1). Notably, more than doubling the data set did not improve the resolution of the symmetrized Hsp104 ATPγS map after refinement (Figure S1), suggesting variability within the data set and/or asymmetry within the hexamers.

Strikingly, the T/T reconstruction without imposed symmetry shows a marked asymmetry in the hexamer, giving the complex a contracted appearance on one side (Figure 5A). On the contracted side, the connection between two of the N domains is broken, and both are pulled down toward the adjacent NBD1 domains. Not all domains are equally well defined or connected to neighboring domains, indicating either mobility of individual domains and/or heterogeneity in the hexamer population. In general, one of the domains per subunit forms a tight contact with at least one neighboring domain in the ring. Raising the density threshold of the map reveals that some domains have less volume than others, indicating regions of high mobility (Figure S3A). The overlay of the 6-fold symmetrized map with the asymmetric Hsp104 T/T reconstruction (Figures 5B and 5C) shows in-plane domain rotations and shifts toward the left-hand or right-hand neighbor in the asymmetric map. When slices of the side view for each subunit are compared (Figure 5D), we observe a gradient of conformations in the AAA+ double layer and the N-terminal densities, ranging from a state resembling the average Hsp104 D/T conformation to one resembling the Hsp104 T/T average. In particular, the subunit exhibiting the strongest density in the AAA+ rings (colored in red) appears to have the most similar conformation to the domains in the symmetrized Hsp104 T/T map. The left-hand neighbor, which exhibits the weakest density in the double layer (colored in yellow), resembles the symmetrized Hsp104 D/T state, in which NBD1 is thought to be posthydrolysis.

In contrast to the asymmetric Hsp104 T/T reconstruction, Hsp104 D/T deviates less from the corresponding symmetrized map (Figures 5F and 5G) and shows a more homogenous density distribution (Figure S3B). However, only four subunits in the hexamer show density connecting the AAA+ domains, and one subunit appears to be entirely detached (colored in red). In the side view slices of each subunit (Figure 5H), we also observe that the height of the double layer varies between individual subunits, giving some NBD pairs a more contracted appearance than others. The two most contracted subunits lie on opposite sites of the hexamer, giving the NBD2 ring a concave shape in side view (Figure S3C).

## Discussion

The reconstructions presented here of Hsp104 hexamers assembled in the presence of ATP and ADP reinforce the notion that the AAA+ domains in Hsp104 form expanded rings enclosing a large central cavity, unlike the classical packing observed in hexameric crystal structures of related proteins (Wendler et al., 2007). Shielding of the coiled coil from antibody binding in the hexameric assembly supports the suggested intercalated location of the domain. Furthermore, we observe large domain movements upon nucleotide hydrolysis, supported by Trp fluorescence measurements, which have not been anticipated from atomic structures of other AAA+ proteins. Our data provide a structural view of the conformational dynamics during Hsp104's ATPase cycle that underlie its remarkable ability to remodel protein aggregates and thereby provide tolerance to a wide variety of proteotoxic stresses.

It should be noted that a different hexamer structure of the closely related ClpB has been published, with a tighter packing of the AAA+ domains and the coiled coil extending radially outward (Lee et al., 2003). Comparison with our maps shows that the outer diameter (∼160 Å) and the strong density features of NBD2 are similar but that there are marked differences in NBD1 and the coiled-coil regions (Figure S6 in Wendler et al., 2007). In different nucleotide states, the ClpB structures appear to differ mainly in the length of the coiled coil visible in the density maps (Lee et al., 2007). We have not observed extended spikes in any of our four independent reconstructions (Wendler et al., 2007 and present data). Refinement of our ATP and ATPγS structures from interchanged or simple geometric starting models yields the original structures (Figure S4). Our maps contain density accounting for all of the domains of Hsp104, including the N-terminal and coiled-coil domains, and conversely, all of the density is accounted for by the subunit domains. In addition, the antibody-binding experiments favor our model of the coiled-coil location. The main experimental difference between the two studies, aside from the species difference, is the use of glutaraldehyde to stabilize the ClpB complex. We have not used any crosslinker in our studies.

### Hsp104 Domain Movements Provide a Structural Basis for N- to C-Terminal Threading

ATP hydrolysis in Hsp104 NBD1 generates a power stroke that displaces the substrate-binding motifs of NBD1 from the N-terminal end of the cavity wall to the center of the cavity. On the other hand, ATP binding to NBD1 moves the tyrosine motifs of NBD2 by ∼20 Å from the center of the cavity toward the C-terminal rim of the cavity. It has been shown that NBD1 activity alone is sufficient to reactivate some aggregated substrates (Doyle et al., 2007). Therefore, it is likely that NBD1 domains can pass through several rounds of ATP binding and hydrolysis without direct coupling to nucleotide consumption in NBD2. As for many AAA+ assemblies, the affinity of Hsp104 for polypeptide substrate is high when ATP is bound (Bosl et al., 2005; Pak et al., 1999; Bolon et al., 2004). Thus, NBD1 tyrosine loops would bind substrate in the ATP-bound state when they are located close to the N termini and lose affinity in the posthydrolysis state, after moving ∼15 Å toward the C-terminal exit of the cavity. Thus, ATP binding and hydrolysis in NBD1 support an N- to C-terminal threading of substrate through the central cavity, a mechanism that has previously been described for ClpB and Hsp104 variants (Weibezahn et al., 2004; Tessarz et al., 2008) but has not been explained structurally so far.

### Weak Domain Contacts Explain Dynamic Properties of Hsp104

Our maps indicate that ATP binding to one AAA+ domain promotes homotypic domain contacts in the other domain. Accordingly, the NBD2 ring is unconnected unless NBD1 is occupied by ATP. ATP binding to NBD2 is necessary for hexamerization of Hsp104 (Schirmer et al., 1998) but does not result in domain interactions within the NBD2 ring in our maps. Therefore, we propose that ATP binding to NBD2 induces a conformational change in Hsp104 that favors oligomerization mediated by NBD1 and the N-terminal domains. Consistent with the large diameter of the hexamer, we observe only limited interdomain contacts between the AAA+ domains in all nucleotide states when compared to other hexameric AAA+ structures. Also, the efficiency of hexamer formation under our EM conditions was best in the presence of ATPγS and declined in the presence of ATP and ADP, which coincides with the extent of domain interactions in the corresponding maps. The weak subunit contacts and large domain movements account for the highly dynamic nature of ClpB/Hsp104 (Werbeck et al., 2008; Schirmer et al., 2001; Haslberger et al., 2008) and distinguish this protein class from other less-dynamic Clp ATPases.

### Model for AAA+ Cooperativity during Substrate Translocation by Hsp104 Hexamers

A wealth of biochemical studies have provided evidence for bidirectional communication between the two AAA+ domains in Hsp104 ( Hattendorf and Lindquist, 2002;Cashikar et al., 2002; Schaupp et al., 2007; Lum et al., 2008), but little is known about the underlying mechanisms. Our model, proposed on the basis of the cryo-EM maps of Hsp104 bound to different nucleotides, accounts for the observed allosteric effects and offers an explanation for the paradoxical observation that NBD2 is a weak ATPase, yet a point mutation in its substrate binding loop abolishes disaggregation activity of Hsp104 (Lum et al., 2004).

**Figure 6:**
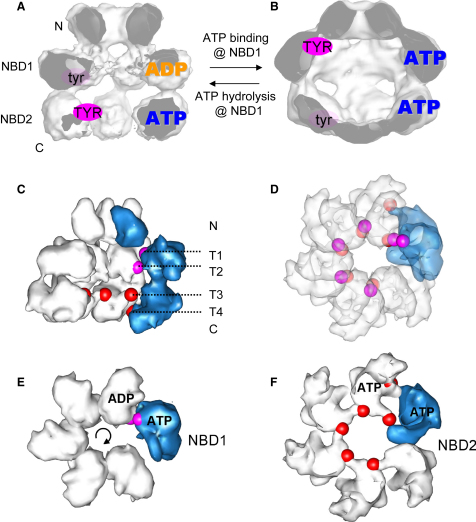
Model for AAA+ Cooperativity and Clockwise ATP Hydrolysis (A) In the presence of ATP, Hsp104^N728A^ is mainly bound to ATP in NBD2, and NBD1 is in a posthydrolysis state (ADP.Pi or ADP). The tyrosine loops in NBD2 are accessible (TYR), whereas the ADP-bound NBD1 is incompetent to bind substrate and Tyr loops are inaccessible (tyr). (B) ATP binding to NBD1 moves the Tyr loops from the center of the cavity to the N-terminal entrance of the cavity, enabling substrate binding in NBD1. NBD2 Tyr loops are rotated downward and toward the right-hand neighbor, where they are occluded in the interface between the subunits. (C–F) Model of a hybrid Hsp104 hexamer consisting of one subunit in the T/T state (blue) and the other five in the D/T state (white). Shown are filtered surface representations of the Hsp104 homology-modeled subunits derived from the fits to the Hsp104^N728A^ ATPγS and ATP maps. In each subunit, Tyr 257 and Tyr 662 are colored in pink and red, respectively. For the side view representation (C), the front two ATP subunits are omitted for clarity. The positions of the tyrosine loops of the T/T subunit (T1, T4) and the D/T state (T2, T3) are indicated. The top view of the AAA+ double layer (D) shows the location of all tyrosine loops in the hexamer by displaying transparent NBDs. The NBD1 (E) and NBD2 (F) rings are displayed as top views.

Starting with the hexameric state of Hsp104^N728A^ in the presence of ATP (D/T) (Figure 6A), we hypothesize that NBD2 is ATP bound, whereas NBD1 is in a posthydrolysis conformation. In our model, the NBD2 Tyr motifs are rotated into the cavity of Hsp104. In this state, NBD2 should be competent to bind substrate. On the other hand, the Tyr loop of NBD1, which has completed a round of ATP hydrolysis, is buried in the interface with the left-hand neighbor in the ring. This model explains why ATP binding to NBD1 is required for Hsp104 to bind substrate (Bosl et al., 2005; Schaupp et al., 2007). When ATP binds to NBD1 (Figure 6B), it enables substrate binding by exposing NBD1-Tyr. Simultaneously, ATP binding to NBD1 rotates the substrate-bound NBD2-Tyr ∼20 Å toward the C-terminal exit of the cavity and toward the right-hand neighbor in the ring. This achieves an extra pulling force by NBD2—comparable to the power stroke in NBD1—without requiring nucleotide hydrolysis in this domain. In addition, it ensures that substrate remains bound to Hsp104 when NBD1 is moving upward to take hold of upstream substrate.

The fit into the Hsp104^N728A^ T/T map places NBD2-Tyr in the interface with the neighboring domain (Wendler et al., 2007). Our fluorescence measurements, in which NBD2-Tyr is replaced by tryptophan, support the fit and indicate that this loop moves into a more hydrophobic environment in the T/T state than in the D/T and D/D states. These homotypic domain contacts in NBD2 might aid substrate release from ATP-bound NBD2, as shown for the disaggregation of some substrates (Doyle et al., 2007). However, the N728A mutation causes loss of thermotolerance in vivo (Hattendorf and Lindquist, 2002), suggesting that ATP hydrolysis in NBD2 is necessary for efficient disaggregation of key substrates. Whether ATP hydrolysis by NBD2 exerts additional pulling force on the substrate and whether it is essential for release of some substrates from NBD2, as suggested by Schaupp et al., 2007, remain to be determined. Owing to instability of the D/D hexamer, data collection for this state was difficult. Therefore, the map is less well defined, and the conclusions drawn from it are less reliable than other results presented here. The fluorescence quenching of NBD2-Trp in the presence of ADP is consistent with multiple possibilities in which the substrate-binding loop is exposed in the D/D state. We present a model based on the observed domain rotation upon ATP hydrolysis in NBD2 (D/D) in our cryo-EM maps, suggesting that the whole subunit disengages from substrate interaction by rotating both Tyr motifs away from the cavity. Thus, we propose that NBD2 aids hexamer formation, supplies crucial substrate interaction sites during the disaggregation activity of Hsp104, and provides a mechanism to release Hsp104 if NBD1 gets stalled. On the other hand, NBD1 activity drives the substrate translocation process by coordinating the Tyr motifs and generating a pulling force in both AAA+ domains, presumably through intervention of the coiled-coil domain.

### Do N Termini Facilitate Substrate Translocation into the Cavity of Hsp104?

The N termini remain connected in all nucleotide states analyzed and form a ring on top of the AAA+ double tier with an opening similar to the pore size of AAA+ crystal structures. Even though the N-terminal ring of the asymmetric Hsp104 ATPγS hexamer appears very distorted, it significantly impairs access to the substrate-binding loops in the NBDs. It has been shown that the N termini are not needed for the normal disaggregation function of Hsp104 (Hung and Masison, 2006). On the other hand, point mutations or inversion of 10 amino acids in the N terminus improve or destroy prion propagation, respectively (Hung and Masison, 2006; Kurahashi and Nakamura, 2007). Therefore, we propose that the N termini of Hsp104 can bind some substrates and that the nucleotide-dependent movements in the AAA+ domains trigger a peristaltic motion that facilitates polypeptide translocation from the N termini to NBD1-Tyr. Despite the finding that ClpB and Hsp104 can thread substrate loops (Haslberger et al., 2008), it remains an open question whether the N-terminal action is sufficient to feed substrate into the Hsp104 cavity or whether the initial binding has to occur via the AAA+ binding sites, inducing the assembly of the hexamer around the substrate.

### Asymmetric Hsp104 Structure Suggests Sequential Processivity

The asymmetric reconstruction of Hsp104^N728A^ ATPγS indicates that at least one NBD1 is captured in the posthydrolysis state, which confirms biochemical results showing that not all subunits in AAA+ hexamers bind and hydrolyze ATP simultaneously (Martin et al., 2005; Briggs et al., 2008), and indicates that ATPγS can be hydrolyzed by Hsp104^N728A^. On the other hand, the asymmetric Hsp104 ^N728A^ ATP map exhibits only one subunit in the hexamer in an upright, ATP-bound state. Thus, we conclude that a non- or poorly hydrolyzable ATP analog is needed to capture the NBD1 ring in an open, upright position, which it does not adopt when ATP can be readily hydrolyzed.

To illustrate a possible mechanism for substrate handover suggested by our results, we created a hybrid model of Hsp104 derived from our fits to the Hsp104^N728A^ ATPγS (T/T) and ATP (D/T) maps, with one subunit in the ATP-bound state in NBD1 and the other five in the ADP state (Figures 6C–6F). In a sequentially firing model, the binding competent NBD1-Tyr loop of the ATP-bound domain binds the substrate at the N-terminal end of the cavity (T1 in Figure 6C), where it is in line with both Tyr loops of the right-hand neighbor in the ring (Figure 6D). ATP hydrolysis in NBD1 results in a rotation of the substrate-bound Tyr loops toward the left-hand neighbor (Figure 6E) and a translocation toward the C-terminal exit of the cavity (T1 to T2 in Figure 6C). Once the substrate reaches the NBD2 ring, the disaggregation activity of each subunit is initiated by the NBD2 power stroke upon ATP binding to NBD1 (T3 to T4 in Figure 6C), which positions polypeptide so that the NBD1-Tyr of the same subunit can continue the translocation (Figures 6F and 6D). Thus, the substrate handover is compatible with a clockwise ATP hydrolysis order in the ring. Given the sparse contacts between subunits in the T/T and D/T states in the hexamer, we favor sequential over stochastic hydrolysis because random ATP binding to NBD1 would either compromise hexamer stability or demand effective communication across several subunits.

The asymmetric maps also explain the qualitative differences between the 6-fold symmetrized reconstructions. The symmetric T/T complex in particular fails to resolve beyond 12 Å even when the data set is increased to ∼23,000 particles, whereas the other reconstructions yield better resolved symmetrized reconstructions with less data. Correspondingly, the T/T complex shows the greatest asymmetry, particularly in the N-terminal ring. The observed distortion suggests that steric hindrance prevents all subunits from simultaneously binding ATP.

### Conclusions

The rotations of the Hsp104 AAA+ domains qualitatively resemble those of the RecA-like gene 4 helicase (Singleton et al., 2000), suggesting a conserved mechanism of ATP hydrolysis that is directed by the coiled-coil insertion in Hsp104 NBD1 to perform its specialized disaggregation function. The large domain movements upon ATP binding and hydrolysis in NBD1 strongly suggest a peristaltic mechanism of substrate threading from the N- to C-terminal side of the complex. Furthermore, we postulate that interdependent actions of NBD1 and NBD2 coordinate substrate translocation, ensuring continuous substrate handover during disaggregation. Our asymmetric reconstructions exclude a concerted hydrolysis in the AAA+ rings, suggesting instead that the observed intersubunit cooperativity generates a sequential firing order. These structural and biochemical results extend and validate the model of Hsp104 structure and function and provide mechanistic information about Hsp104's disaggregative AAA+ motor activity.

## Experimental Procedures

### Cryo-Electron Microscopy

Hsp104^N728A^ was purified as described in Wendler et al. (2007) and diluted to a final concentration of 0.3 mg/ml in 20 mM HEPES (pH 7.5), 20 mM NaCl, 10 mM MgCl_2_, 1 mM DTT, and 2–5 mM nucleotide. A 3.5 μl sample of the solution was applied to glow discharged, lacey carbon film on 300 mesh copper grids. After 30 s, excess solution was blotted, and the grid was flash-frozen in liquid ethane. Cryo-EM was carried out on a Tecnai F20 FEG operated at 200 kV under low-dose conditions. Images were taken at a magnification of 50,000× with defocus ranging from 1.0 to 4.0 μm (ATPγS), from 1.5 to 3.5 μm (ATP), and from 1.5–5.2 μm (ADP) (Figure S5).

### Image Processing and 3D Reconstruction

Micrographs were digitized on a SCAI microdensitometer (Zeiss) at 1.4 Å per pixel at the specimen level. A total of 23,632 (Hsp104^N728A^ ATPγS), 11,128 (Hsp104^N728A^ ATP), and 2379 particles (Hsp104 ^N728A^ ADP) were manually selected from 95, 43, and 36 micrographs, respectively, with the MRC program Ximdisp (Crowther et al., 1996). The 6-fold symmetrized maps of Hsp104^N728A^ ATP and Hsp104^N728A^ ADP were obtained from 4046 and 2379 particles, respectively. Phase correction for effects of the contrast transfer function, binning to 2.8 Å per pixel, as well as generation of the initial map by angular reconstitution and refinement to the final reconstruction were performed independently for each data set as described in Wendler et al. (2007). The final reconstructions contained ∼75% of the particles of each data set, resulting in structures at resolutions of 11.5 Å (ATP) and 12.8 Å (ADP), estimated by Fourier shell correlation with a 0.5 correlation cutoff and loose masking. Without masking, the resolutions were 16.4 and 16.8 Å for Hsp104^N728A^ ATP and Hsp104^N728A^ ADP, respectively (Figure S1).

For the reconstructions without imposed symmetry, the ATPγS and ATP data sets were extended to the totals mentioned above, and particle orientations were determined in two rounds of projection matching using the 6-fold symmetrized map as a starting model. Subsequently, particle orientations were refined with projection matching in SPIDER without imposed symmetry. After 22 cycles of projection matching, decreasing the angular step to 2°, ∼90% of the assigned angles were stable for the ATPγS data set (Figure S6). The angles in ATP data set stabilized after nine rounds with an 8° angular step. The final resolutions are at 11.5 Å (15.3 Å without mask) for the ATPγS reconstruction and 14.1 Å (20.3 Å without mask) for the ATP reconstruction, estimated by Fourier shell correlation with a 0.5 correlation cutoff (Figure S1).

### Atomic Structure Fitting

Docking was done with the Hsp104 homology model as described in Wendler et al. (2007). The AAA+, coiled-coil, and N-terminal domains were fit manually as rigid bodies into the EM densities with PYMOL (http://www.pymol.org). All figures were produced with the UCSF Chimera package (Pettersen et al., 2004) except for Figure 3, which was done with PYMOL.

### Dot Blots

Hsp104 (2 μM) was dialyzed into 20 mM HEPES-KOH (pH 7.5), 200 mM KCl, 10 mM MgCl_2_, 2 mM EDTA, and 2 mM DTT ± 10 mM ATPγS. Under these conditions, Hsp104 is monomeric in the absence of nucleotide and hexameric in the presence of ATPγS (Parsell et al., 1994). Between 0.25 and 1 μg of monomeric or hexameric Hsp104 was applied to nitrocellulose and blocked with 10% nonfat milk in Tris-buffered saline (TBS) at 4°C for 1 hr. Tween 20 was omitted from all buffers because it interferes with Hsp104 hexamerization. Blots were then probed for 10 min at 4°C with monoclonal antibodies that recognize either the N-terminal domain (28B), middle domain (4G10, 4B, and 17B), or C-terminal domain (27B) of Hsp104 (Cashikar et al., 2002). Membranes were washed, incubated with horseradish peroxidase-conjugated anti-mouse IgG (Sigma), and processed for chemiluminescence.

### Tryptophan Fluorescence

The Hsp104^Y662W:N728A^ mutant was generated by Quikchange mutagenesis (Stratagene). Hsp104^Y662W^ or Hsp104^Y662W:N728A^ were buffer exchanged using Bio-Gel P6 columns into 20 mM HEPES-KOH (pH 7.5), 20 mM NaCl, 10 mM MgCl_2_, and 1 mM DTT. Under these conditions, Hsp104 is hexameric even in the absence of nucleotide (Hattendorf and Lindquist, 2002). Any particulate matter was removed by centrifugation at 16,100 × g for 10 min. Hsp104^Y662W^ or Hsp104^Y662W:N728A^ (2 μM) were then incubated in the absence or presence of ATPγS, ATP, or ADP (1 mM) for 10 min at 25°C. Tryptophan fluorescence at 346 nm (10 nm bandwidth) was measured after excitation at 295 nm (5 nm bandwidth) with a Safire^2^ plate reader (Tecan). The change in fluorescence (ΔF) was determined by comparing the fluorescence after 10 min (F) to the starting fluorescence (F_0_; before nucleotide addition), such that ΔF = (F/F_0_)−1.

## Accession Numbers

The cryo-EM maps have been deposited in the 3D-EM database (http://www.emdatabank.org) with accession codes EMD-1599 (Hsp104 ^N728A^ ADP), EMD-1600 (Hsp104 ^N728A^ ATP C6), EMD-1601 (Hsp104 ^N728A^ ATP C1), and EMD-1602 (Hsp104 ^N728A^ ATPγS C1).

## Supplemental Data

Document S1. Six Figures

Movie S1. Alternation of TT and DT States of Hsp104 Seen in Cut-Away Side View

Movie S2. Alternation of DT and DD States of Hsp104 Seen in Cut-Away Side View

Movie S3. Alternation of TT and DT States of Hsp104 Seen in Side View

Movie S4. Alternation of DT and DD States of Hsp104 Seen in Side View The movies show sequences of surface-rendered images of Hsp104 cryo-EM reconstructions in complex with different nucleotides. They should be viewed as a repeating loop and give the impression of domain movements occurring during the ATPase cycle.
