# Supplementary material for: Motor Mechanism for Protein Threading through Hsp104
Source: Mol Cell. 2009 Apr 10;34(1):81–92. doi: 10.1016/j.molcel.2009.02.026 (PMC2689388; doi:10.1016/j.molcel.2009.02.026)
Supplement: Document S1. Six Figures [file mmc1.pdf]

Molecular Cell, ***Volume 34***

Supplemental Data

Motor Mechanism for Protein Threading through Hsp104

**Petra Wendler, James Shorter, David Snead, Celia Plisson,
Daniel K. Clare, Susan Lindquist, and Helen R. Saibil**

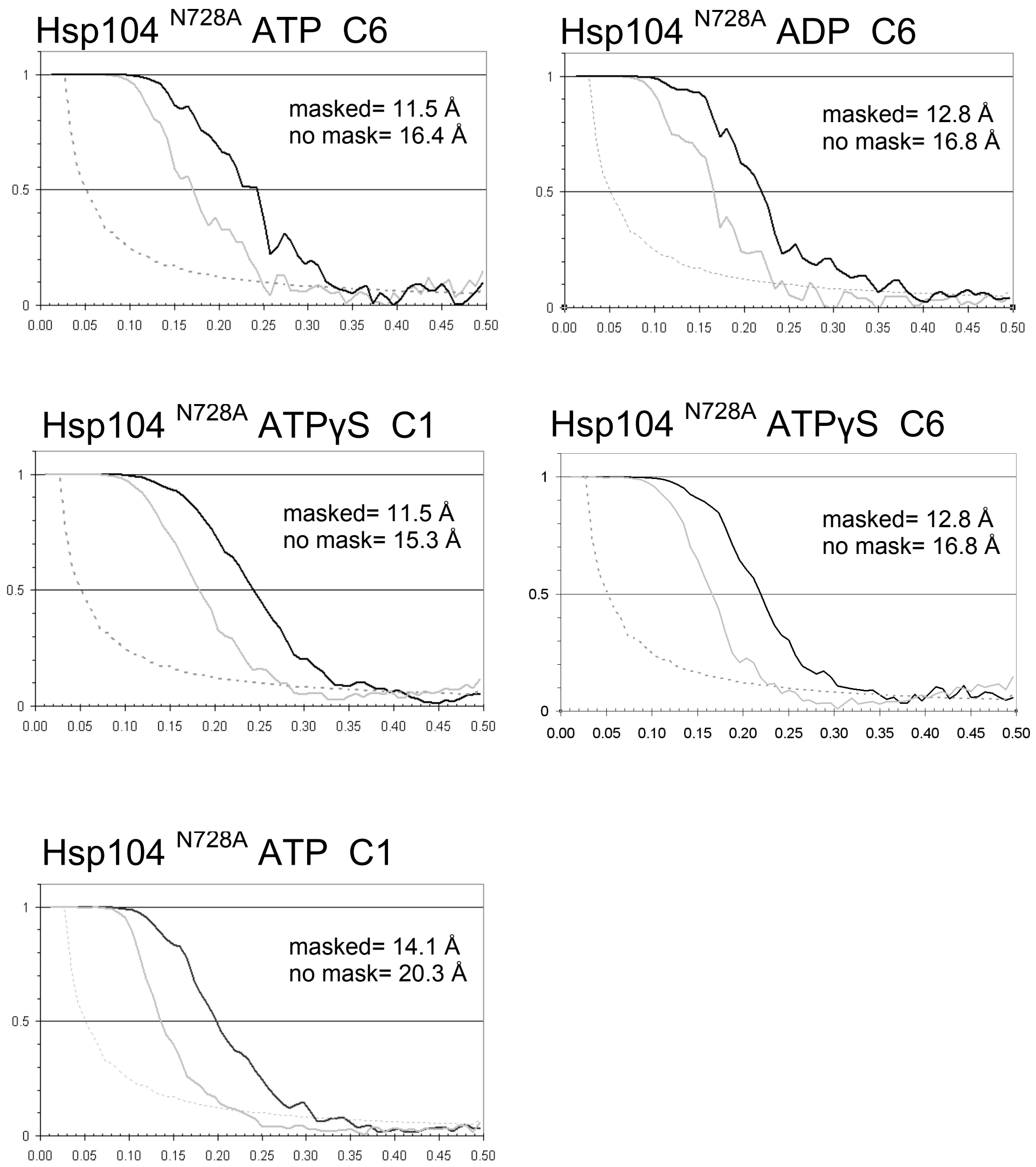

Figure S1. Fourier Shell Correlation Analysis

Fourier shell correlation curves of all Hsp104 maps discussed in this work calculated without mask (grey) and with masking (black). The resolution measured using the 0.5 cut-off criterion is given as an inset in each diagram. The sample and symmetry applied during refinement are given above each plot. The graphs show the Fourier Shell Correlation plotted against spatial frequency [1/Å].

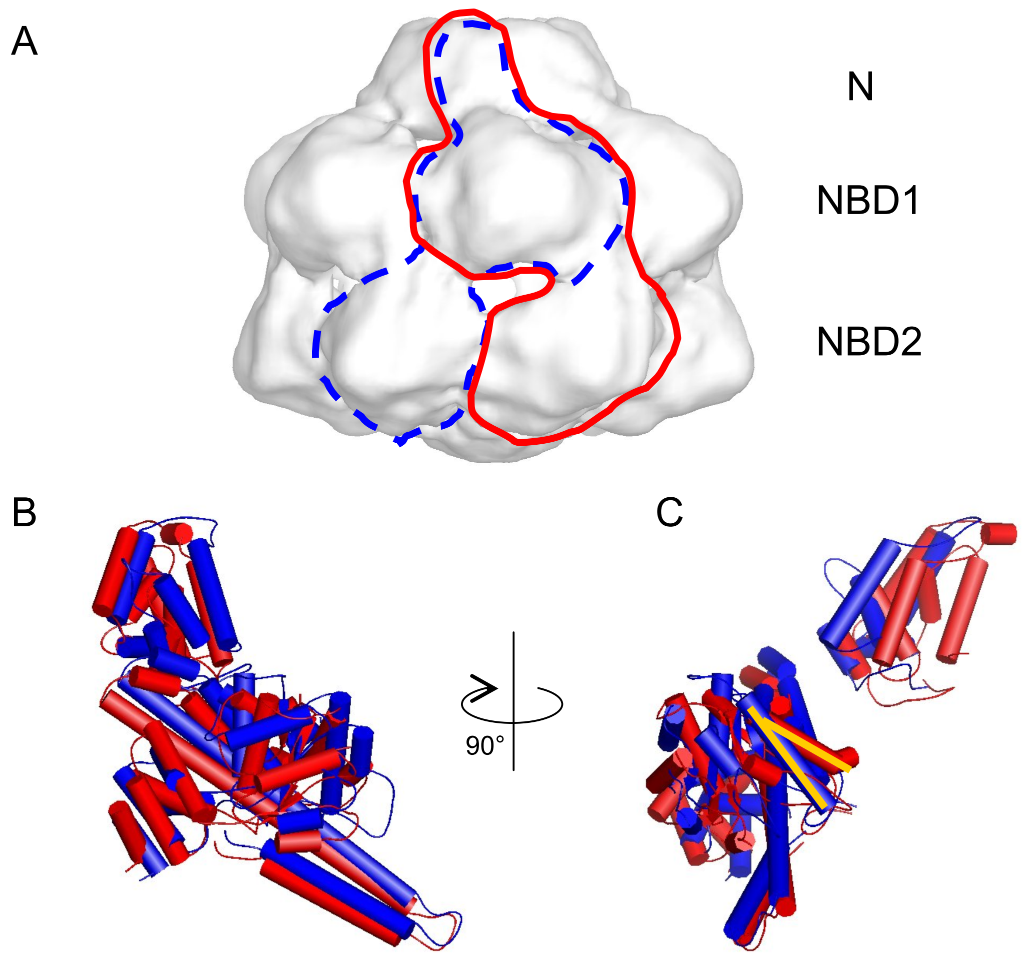

Figure S2. Altered Fit for Hsp104^{N728A} ATPγS

A) The red outline shows the new and the dashed blue outline shows the previously proposed subunit connectivity between NBD1 and NBD2 in Hsp104.

B) Rigid body fit of the N, NBD1 and coiled coil domains in the side view orientation shown in A, and (C), a 90° rotated view. The red structure consists of the ClpB x-ray structure (N-terminus) and the Hsp104 homology model (NBD1) and shows the new fit. The blue structure is as published in Wendler *et al.*, 2007 (Atypical AAA+ subunit packing creates an expanded cavity for disaggregation by the protein-remodeling factor Hsp104. Cell *131*, 1366-1377). The yellow lines in C mark the same alpha helix in the two fits.

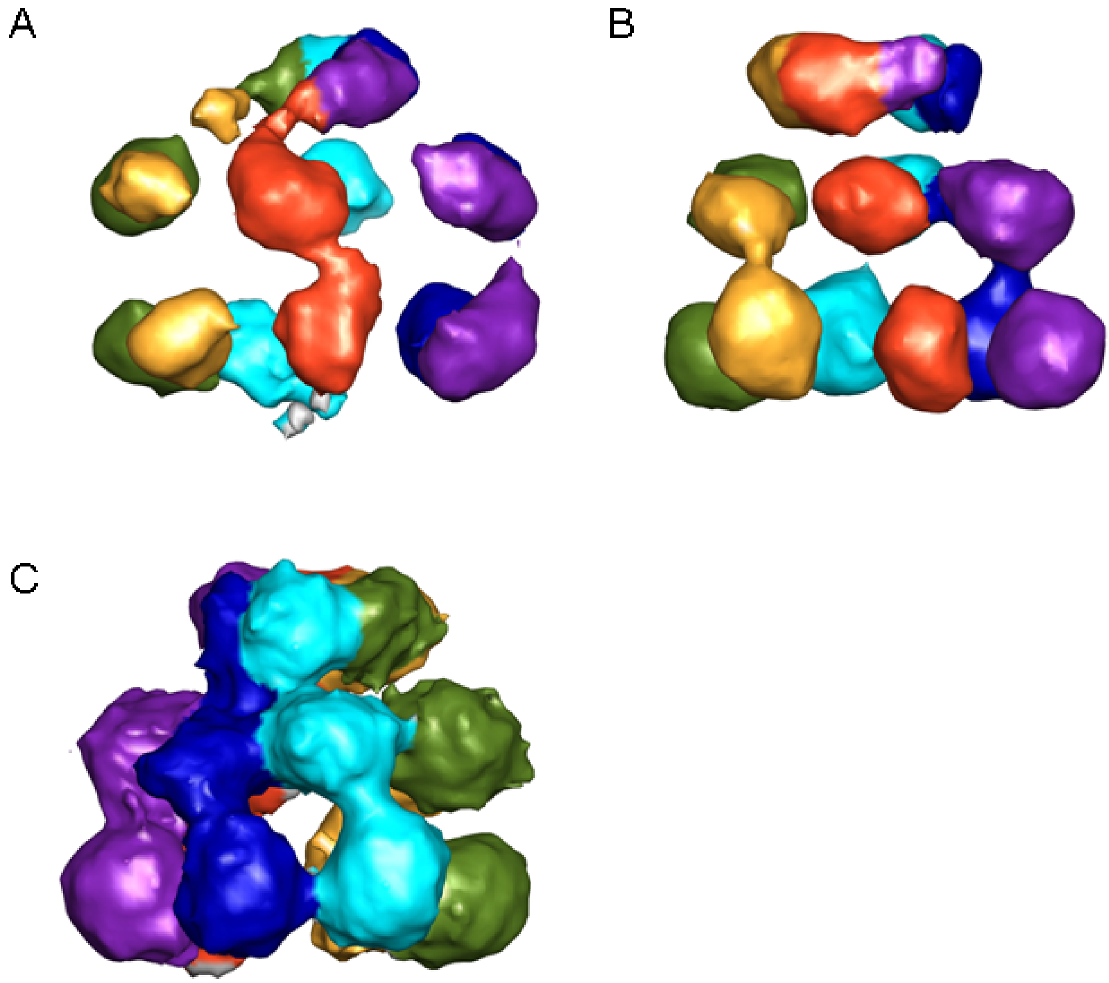

Figure S3. Asymmetric Hsp104 Reconstructions

A) High threshold rendered image of Hsp104 ^{N728A} ATPγS. Orientation and color code as in Figure 4A.

B) High threshold rendered image of Hsp104 ^{N728A} ATP. Orientation and color code as in Figure 4E.

C) Side view of Hsp104 ^{N728A} ATP as in Figure 4E, but rotated by ~160° around the 6-fold axis.

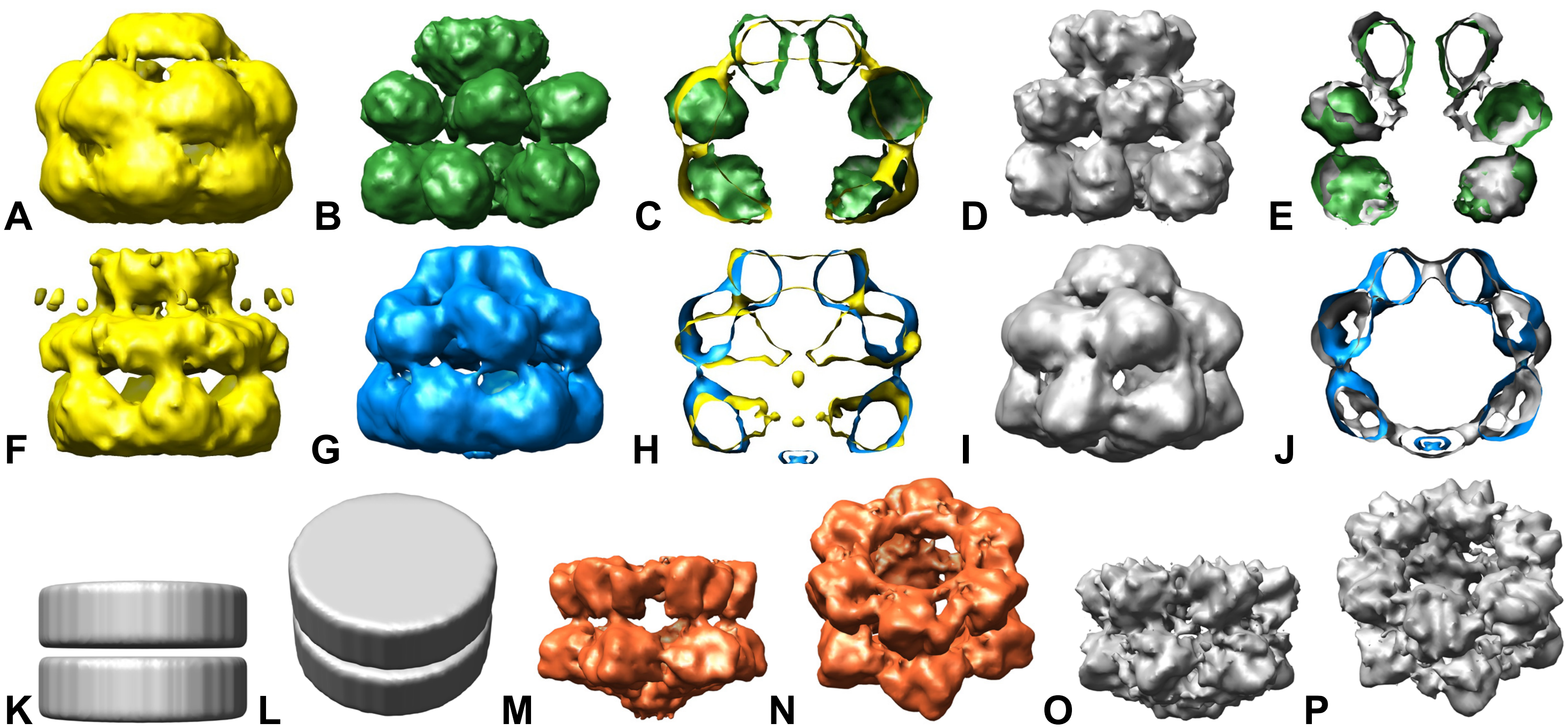

Figure S4. Refinement from Exchanged References
A) Starting model generated from the ATPγS dataset. **B**) 3D reconstruction of ATP dataset after refinement by projection matching (~65% stable angles; 3° angular step) from the starting model in A. **C**) Overlay of map sections from A and B. **D)** Original D/T map refined from F (~90% stable angles; 3° angular step) by projection matching. **E**) Overlay of map sections from B and D. **F**) Starting model generated from the ATP dataset. **G**) 3D reconstruction of ATPγS dataset after refinement by projection matching (~70% stable angles; 3° angular step) from the starting model in F. **H**) Overlay of map sections from F and G. **I**) Original T/T map refined from A (~90% stable angles; 2° angular step) by projection matching. **J**) Overlay of map sections from G and I. **K, L)** Side and tilted views of solid stacked disk starting model. **M, N)** Same views of Hsp104 ΔN map refined by projection from the solid disks (~75% stable angles; 3° angular step). **O, P)** Same views of the original Hsp104 ΔN structure published in Wendler *et al.*, 2007.

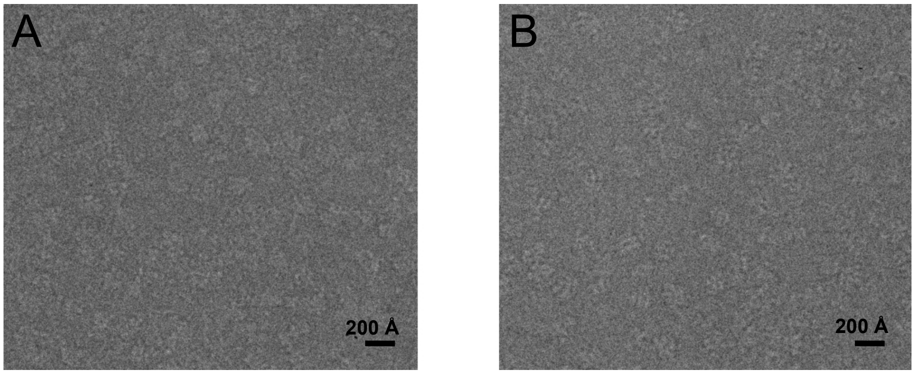

Figure S5. Raw Images of Hsp104 Complexes in the Presence of ATP and ADP

A) Cryo EM raw image of Hsp104 N728A in the presence of 5 mM ADP

B) Cryo EM raw image of Hsp104 N728A in the presence of 5 mM ATP

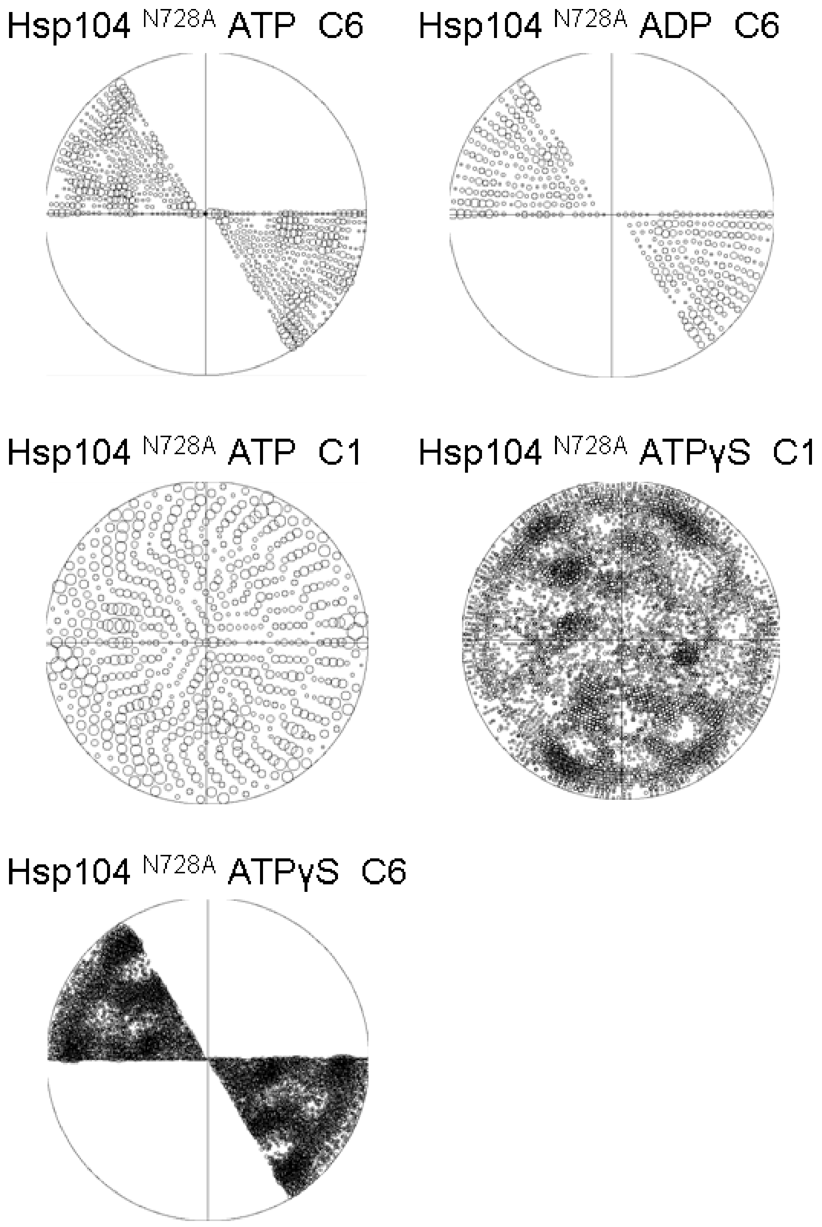

Figure S6. Euler Angle Distribution

Shown is the Euler angle distribution for all Hsp104 datasets used in this work after refinement with projection matching. Analyzed dataset and symmetry applied during refinement are given above each map. Discrete directions with 3° ($Hsp104^{N728A}$ ATP C6), 4° ($Hsp104^{N728A}$ ADP C6), 8° ($Hsp104^{N728}$ ATP C1), 2° ($Hsp104^{N728A}$ ATPγS C1) and 1° ($Hsp104^{N728A}$ ATPγS C6) azimuthal equidistant angular spacing are represented by a circle, whose size corresponds to the number of particles matching that direction.
