# Supplementary figures and images for: Motor Mechanism for Protein Threading through Hsp104
Source: Mol Cell. 2009 Apr 10;34(1):81–92. doi: 10.1016/j.molcel.2009.02.026 (PMC2689388; doi:10.1016/j.molcel.2009.02.026)

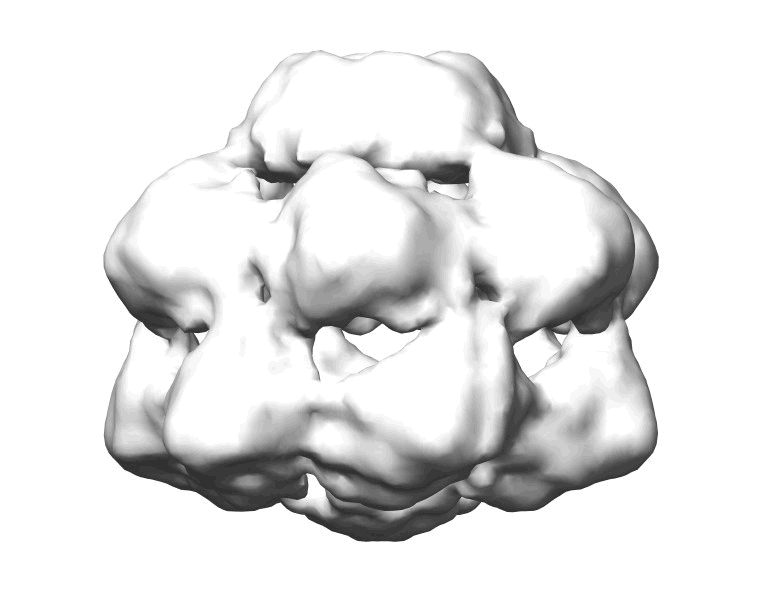

Supplement: Movie S1. Alternation of TT and DT States of Hsp104 Seen in Cut-Away Side View [file mmc2.gif]

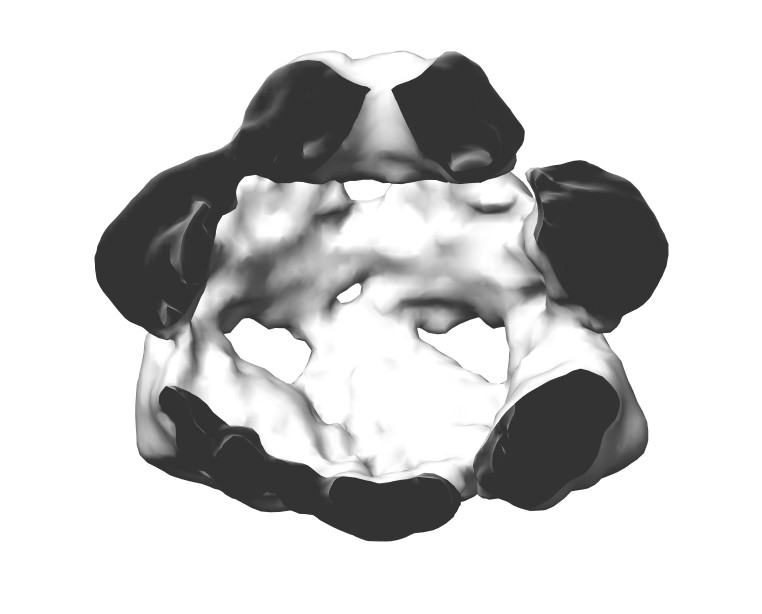

Supplement: Movie S2. Alternation of DT and DD States of Hsp104 Seen in Cut-Away Side View [file mmc3.gif]

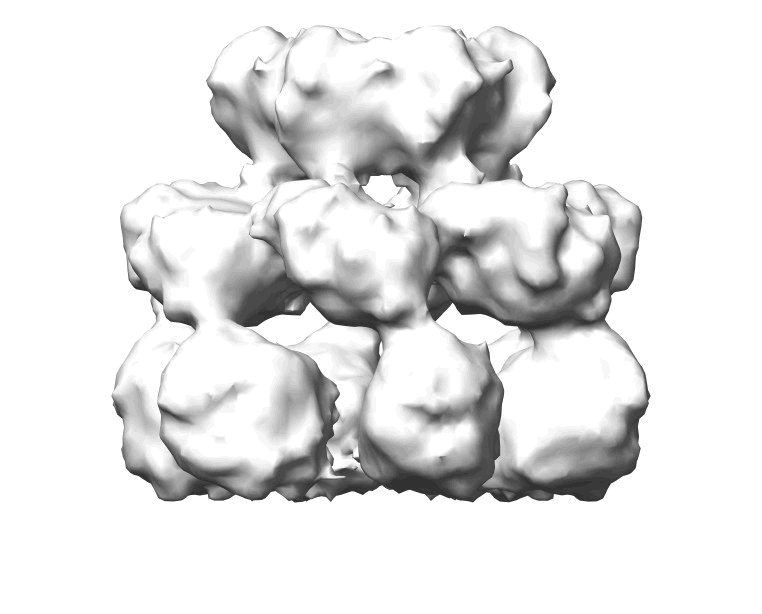

Supplement: Movie S3. Alternation of TT and DT States of Hsp104 Seen in Side View [file mmc4.gif]

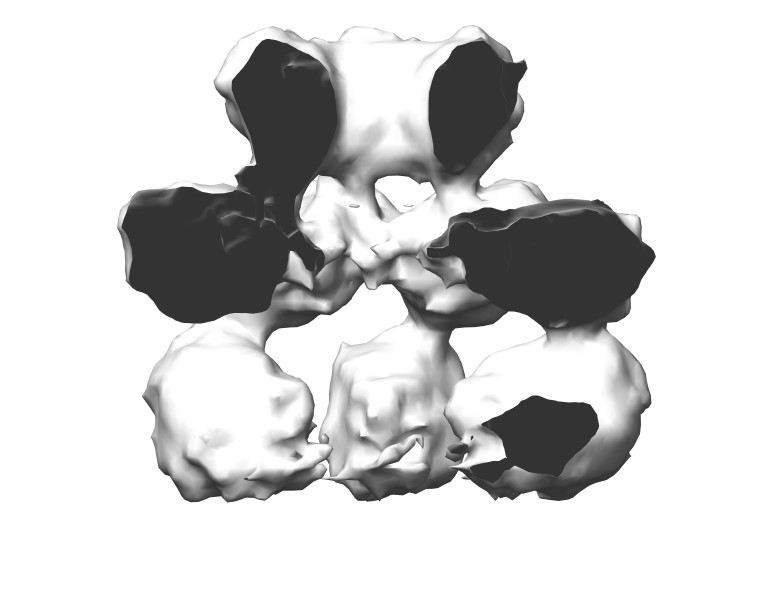

Supplement: Movie S4. Alternation of DT and DD States of Hsp104 Seen in Side View — The movies show sequences of surface-rendered images of Hsp104 cryo-EM reconstructions in complex with different nucleotides. They should be viewed as a repeating loop and give the impression of domain movements occurring during the ATPase cycle. [file mmc5.gif]
